# Supplementary material for: Ingestion of artificial sweeteners leads to caloric frustration memory in Drosophila
Source: Nat Commun. 2017 Nov 27;8:1803. doi: 10.1038/s41467-017-01989-0 (PMC5703724; doi:10.1038/s41467-017-01989-0)
Supplement: Supplementary file 1 — Supplementary Information [file 41467_2017_1989_MOESM1_ESM.pdf]

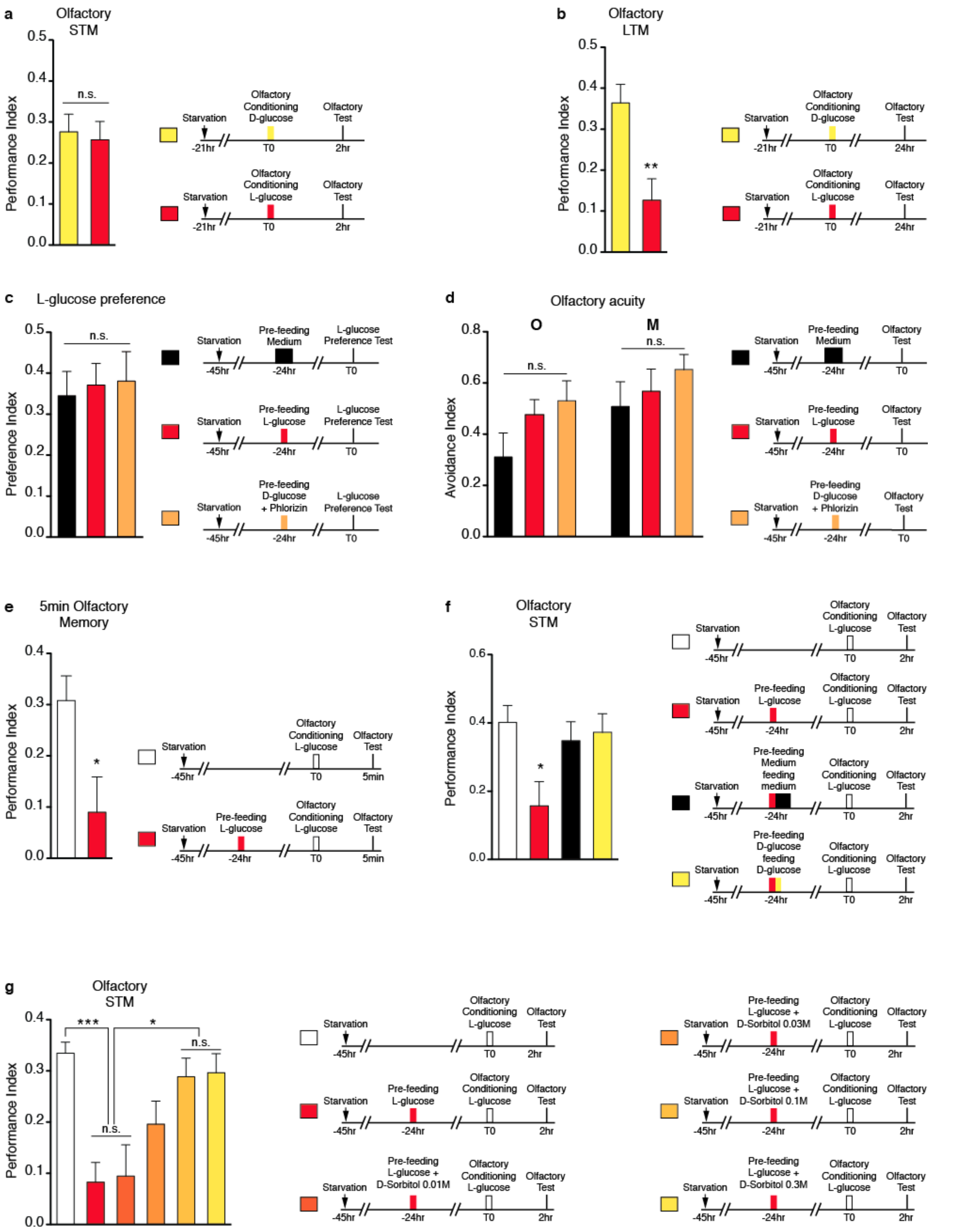

Supplementary Figure 1: Energy is required for LTM formation. a Flies conditioned on either L- or D-glucose display similar olfactory memory scores 2 hr after training *(t-test*, t_{28} = 0.311; p = 0.757; n = 15). **b** Flies conditioned on energetic D-glucose display higher olfactory long-term memory scores than L-glucose conditioned flies (*t-test*, t_{31} = 3.440; p = 0.001; n =15). **c** Pre-feeding flies with L-glucose or a mixture of D-glucose + phlorizin 24 hr before an L-glucose preference test does not affect sugar preference in comparison to flies pre-fed with medium ($F_{(2,49)}$ = 0.09; p = 0.913; n ≥ 16). **d** Normal

olfactory acuity was observed in flies pre-fed with regular medium, L-glucose, or a mixture of D-glucose + phlorizin in response to octanol ($F_{(2,41)}$ = 2.173, p = 0.127, n = 14) and methylcyclohexanol ($F_{(2,41)}$ = 0.773, p = 0.468, n = 14). **e** Flies pre-fed and conditioned with L-glucose display a significantly lower memory score as compared to non-pre-fed flies, when the olfactory memory test is performed 5 min after training (t-test, t_{17} = 2.636; p = 0.017; $n \geq 9$). **f** Both non-pre-fed flies and flies pre-fed with L-glucose and re-fed on D-glucose or classical medium displayed significantly higher STM scores as compared to L-glucose pre-fed flies ($F_{(3,46)}$ = 3.445; p = 0.248; $n \geq 10$). **g** Complementing L-glucose pre-feeding with increasing concentrations of energetic but tasteless D-sorbitol progressively inhibits CFM formation ($F_{(6,161)}$ = 8.6; $p < 0.0001$; $n \geq 18$; $p > 0.999$ in *post-hoc* comparison between flies pre-fed with L-glucose and flies pre-fed with L-glucose and D-sorbitol 0.01M, $p > 0.999$ in *post-hoc* comparison between flies pre-fed with L-glucose and D-sorbitol 0.1M and flies pre-fed with L-glucose and D-sorbitol 0.3M). Means are ± SEM; statistical tests: t-test and one-way ANOVA; n.s.: $p \geq 0.05$; *$p < 0.05$; **$p < 0.01$; ***$p < 0.001$ in comparison between two groups for t-test and in post hoc comparisons with other groups for ANOVA.

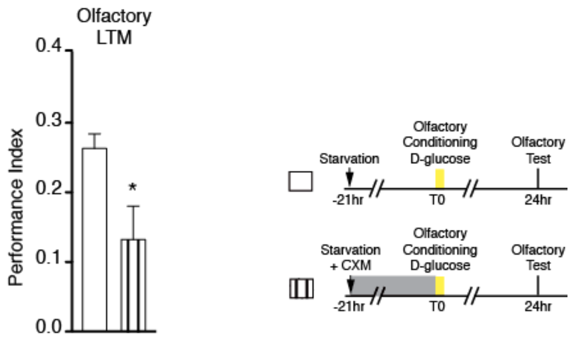

Supplementary Figure 2: Twenty-four-hour memory formed after conditioning with D-glucose is protein synthesis-dependent. Treating flies with cycloheximide protein synthesis inhibitor (CXM) impairs 24-hr memory in flies conditioned with D-glucose (*t*-test, $t_{37} = 2.446$; $p = 0.019$; $n \geq 19$). Means are ± SEM; statistical test: *t*-test; n.s.: $p \geq 0.05$; *$p < 0.05$; in comparison between two groups.

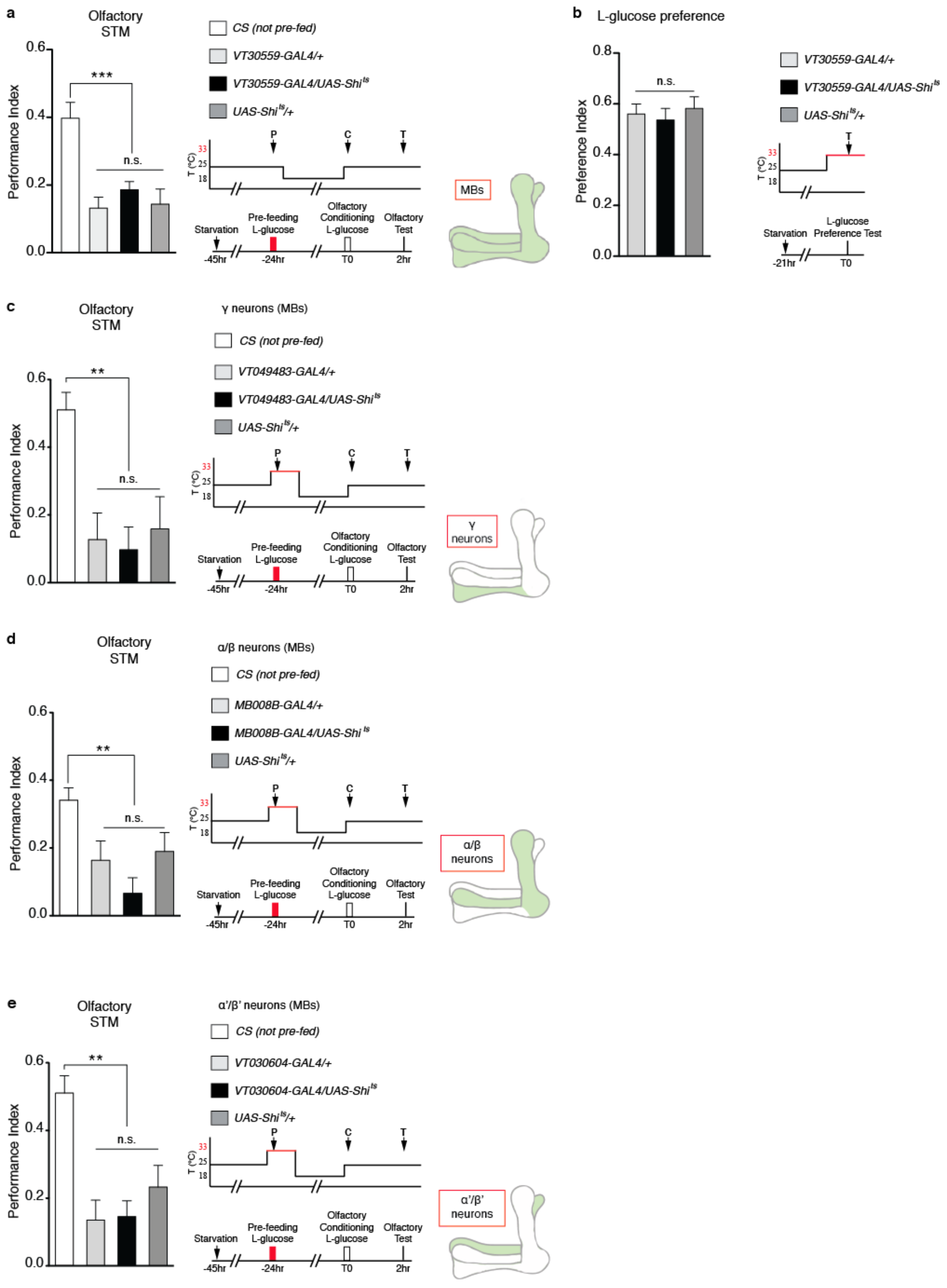

Supplementary Figure 3: Unlike inhibition of the complete MB, inhibiting a sub-population of MB neurons does not impair CFM. a Shi^{ts} expression in MB neurons under VT30559-GAL4 does not impair CFM at the permissive temperature ($F_{(3,63)}$ = 8.966; p < 0.001; n ≥ 15; p = 0.708 in *post-hoc* comparison between *UAS-Shi^{ts}/+* and *VT30559-GAL4/UAS-Shi^{ts}*, p = 0.996 in *post-hoc* comparison between *UAS-Shi^{ts}/+* and *VT30559-GAL4/+*, p = 0.846 in *post-hoc* comparison between *VT30559-GAL4/UAS-Shi^{ts}* and *VT30559-GAL4/+*). **b** At the restrictive temperature, the L-glucose response of flies expressing Shi^{ts} in MB neurons does not differ from controls ($F_{(2,71)}$ = 0.273; p > 0.05; n = 24). **c** Blocking MB γ neurons with *VT049483-GAL4* during and after L-glucose pre-feeding does not abolish CFM ($F_{(3,39)}$ = 8.559; p = 0.0002; n ≥ 8; p = 0.990 in *post-hoc* comparison between *VT49483-GAL4/+* and *VT49483-GAL4/UAS-Shi^{ts}*, p = 0.991 in *post-hoc* comparison between *UAS-Shi^{ts}/+* and *VT49483-GAL4/+*, p = 0.933 in *post-*

hoc comparison between *VT49483-GAL4/UAS-Shi*^{ts} and *UAS-Shi*^{ts}*/+*). **d** Blocking MB α/β neurons with *MB008B-GAL4* during and after L-glucose pre-feeding does not abolish CFM ($F_{(3,48)}$ = 6.962; $p < 0.0001$; $n \geq 10$; p = 0.528 in *post-hoc* comparison between *MB008B-GAL4/+* and *MB008B-GAL4/UAS-Shi*^{ts}, p = 0.322 in *post-hoc* comparison between *UAS-Shi*^{ts}*/+* and *MB008B-GAL4/UAS-Shi*^{ts}, p = 0.984 in *post-hoc* comparison between *MB008B-GAL4/+* and *UAS-Shi*^{ts}*/+*). **e** Blocking MB α'/β' neurons with *VT030604-GAL4* during after L-glucose pre-feeding does not abolish CFM ($F_{(3,31)}$ = 12.15; $p < 0.0001$; $n \geq 6$; p = 0.999 in *post-hoc* comparison between *VT30604-GAL4/+* and *VT30604-GAL4/UAS-Shi*^{ts}, p = 0.688 in *post-hoc* comparison between *UAS-Shi*^{ts}*/+* and *VT30604-GAL4/+*, p = 0.754 in *post-hoc* comparison between *VT30604-GAL4/UAS-Shi*^{ts} and *UAS-Shi*^{ts}*/+*). Means are ± SEM; one-way ANOVA; n.s.: $p \geq 0.05$; **$p < 0.001$ in post hoc comparisons with both parental controls.

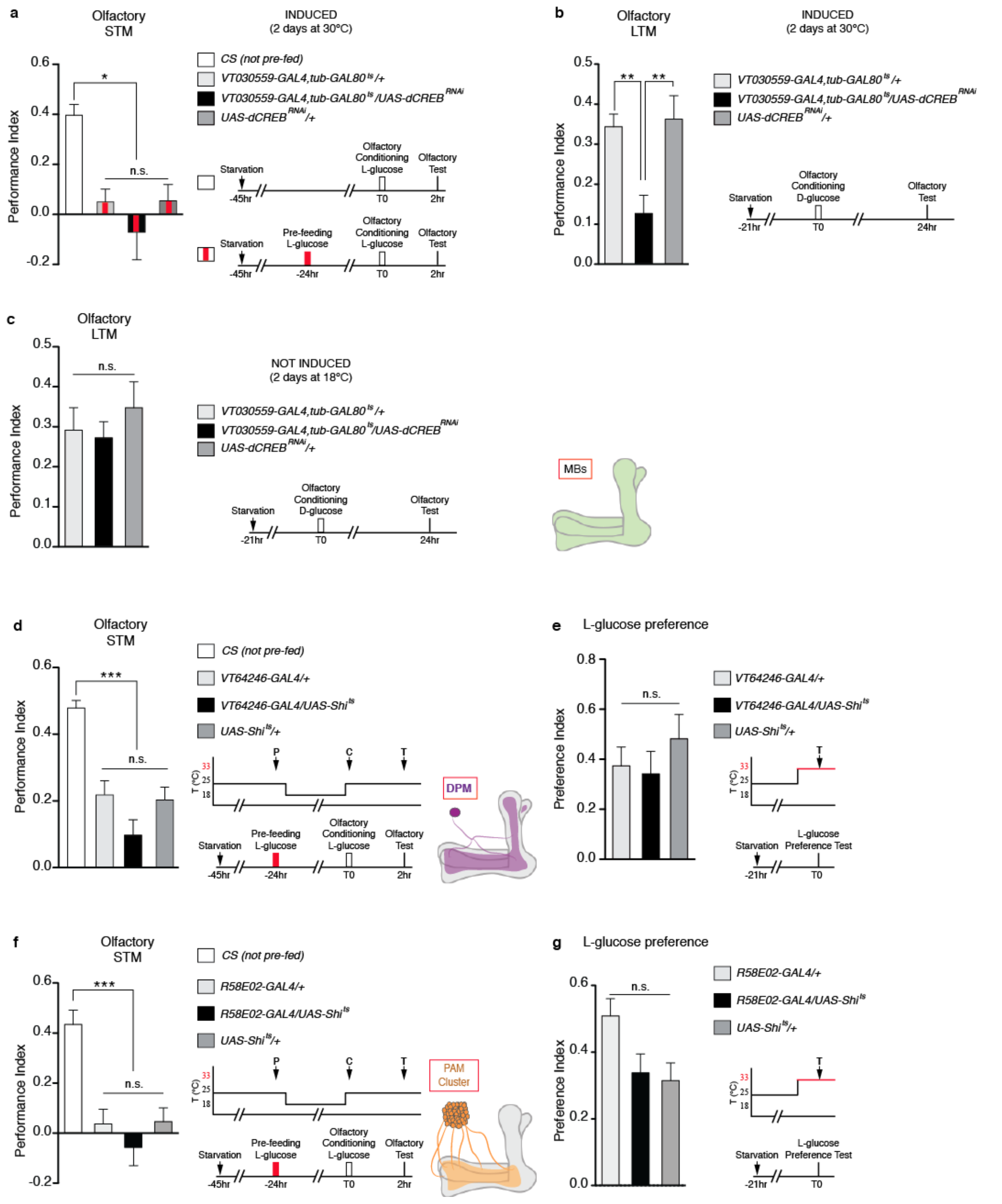

Supplementary Figure 4. Experimental controls for CFM impairment by MB, DPM and PAM neuron blockade. a CFM was not impaired by RNAi inhibition of dCREB in MBs of Adult flies conditioned with L-glucose ($F_{(3,32)}$ = 5.542; p = 0.039; $n \geq 6$; p = 0.658 in *post-hoc* comparison between *VT030559-GAL4,tub-GAL80*^{ts}*/+* and *VT030559-GAL4,tub-GAL80*^{ts}*/UAS-dCREB*^{RNAi}, p > 0.999 in *post-hoc* comparison between *VT030559-GAL4,tub-GAL80*^{ts}*/+* and *UAS-dCREB*^{RNAi}*/+*, p = 0.632 in *post-hoc* comparison between *VT030559-GAL4,tub-GAL80*^{ts}*/UAS-dCREB*^{RNAi} and *UAS-dCREB*^{RNAi}*/+*). **b** LTM was impaired by RNAi inhibition of dCREB in MBs of adult flies conditioned with D-glucose ($F_{(2,50)}$ = 8.166; p < 0.001; $n \geq 17$). **c** After 2 days at 18°C, control tub-GAL80^{ts};*VT030559-GAL4/dCREB*^{RNAi} flies displayed normal LTM ($F_{(2,32)}$ = 0.487, p = 0.618, $n \geq 10$). **d** Shi^{ts} expression in DPM neurons with VT64246-GAL4 control does not impair CFM ($F_{(3,101)}$ = 0.290; p < 0.001; $n \geq 19$; p = 0.221 in *post-hoc* comparison between *VT64246-GAL4/+* and *VT64246-GAL4/UAS-Shi*^{ts}, p = 0.992 in *post-hoc* comparison between *VT64246-GAL4/+* and *UAS-Shi*^{ts}*/+*, p = 0.144 in *post-hoc* comparison between *VT64246-GAL4/UAS-Shi*^{ts} and *UAS-Shi*^{ts}*/+*). **e** At the restrictive temperature, the L-glucose response of flies expressing Shi^{ts} in

DPM neurons does not differ from that of control genotypes ($F_{(2,54)}$ = 0.686; $p > 0.05$; $n \geq 17$). **f** Shi^{ts} expression in PAM neurons with R58E02-GAL4 control does not impair CFM ($F_{(3,69)}$ = 8.589; $p < 0.001$; $n \geq 11$; p = 0.685 in *post-hoc* comparison between *UAS-Shi*^{ts}*/+* and *R58E02-GAL4/UAS-Shi*^{ts}, p = 0.999 in *post-hoc* comparison between *R58E02-GAL4/+* and *UAS-Shi*^{ts}*/+*, p = 0.616 in *post-hoc* comparison between *R58E02-GAL4/UAS-Shi*^{ts} and *R58E02-GAL4/+*). **g** At the restrictive temperature, the L-glucose response of flies expressing Shi^{ts} in PAM neurons does not differ from that of control genotypes ($F_{(2,49)}$ = 3.891; $p > 0.05$; $n \geq 16$). Means are ± SEM; statistical test: one-way ANOVA; n.s.: p ≥ 0.05; ***p < 0.001 in post hoc comparisons with both parental controls.

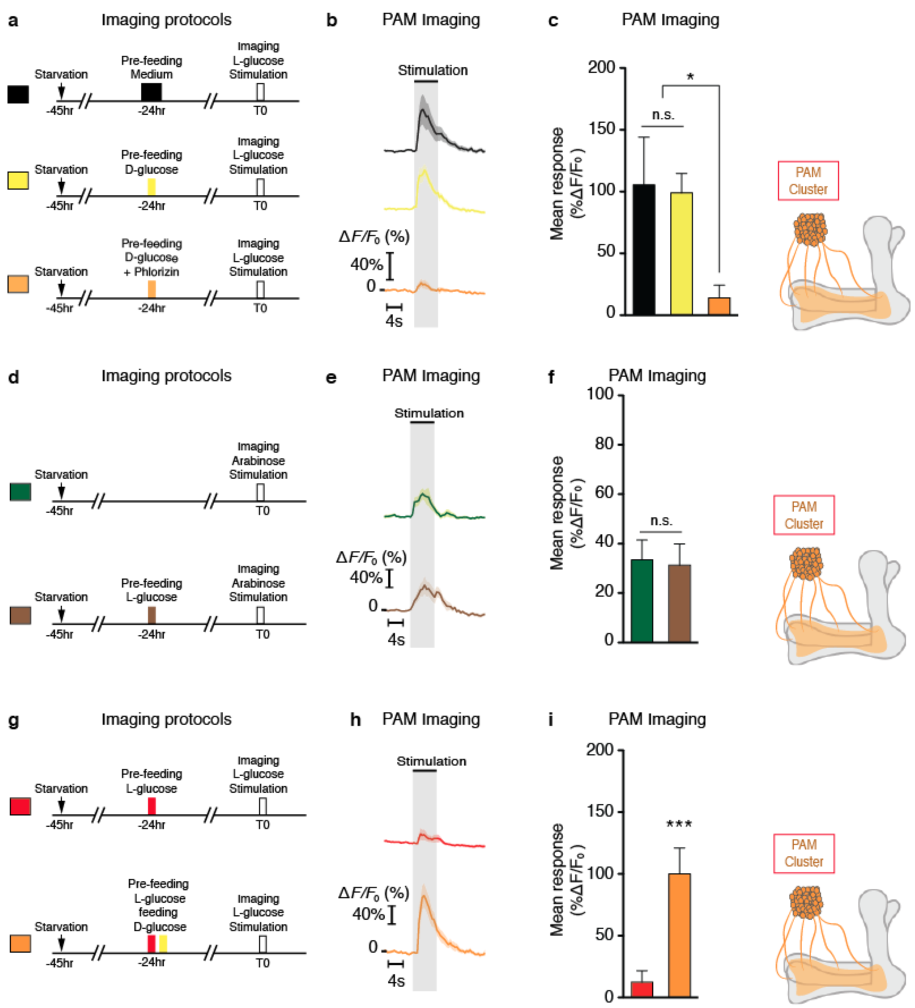

Supplementary Figure 5. Additional experiments for PAM Imaging. a Pre-feeding protocol used before the imaging experiment: flies were pre-fed with either classical medium for 30 min, D-glucose for 1 min, or a mixture of D-glucose and phlorizin 24 hr before L-glucose stimulation under the microscope. **b** Time course response of PAM neurons. $n \geq 8$. Black bar: stimulus presentation. **c** Average response to L-glucose. Flies pre-fed on a mixture of D-glucose and phlorizin displayed a significantly lower response in comparison to flies pre-fed on medium and D-glucose pre-fed flies ($F_{(2,27)} = 5.783$; $p = 0.008$; $n \geq 8$; $p = 0.978$ in *post-hoc* comparison between flies pre-fed with medium and flies pre-fed with D-glucose). **d** Pre-feeding protocol used before the imaging experiment: flies were pre-fed or not with L-glucose for 1 min, 24 hr before arabinose stimulation under the microscope. **e** Time course of response. $n \geq 9$. Black bar: stimulus presentation. **f** Average response to arabinose. Flies pre-fed on L-glucose displayed an equivalent response in comparison to non-pre-fed flies (*t*-test, $t_{17} = 0.18$; $p = 0.852$; $n \geq 10$). **g** Pre-feeding protocol used before the imaging experiment: flies were pre-fed either L-glucose for 1 min or L-glucose for 1 min and immediately fed on D-glucose for 1 min, 24 hr before L-glucose stimulation under the microscope. **h** Time course response of PAM neurons. $n \geq 8$. Black bar: stimulus presentation. **i**, Average response to L-glucose. Flies pre-fed with L-glucose and re-fed on D-glucose displayed a significantly higher response in comparison to L-glucose pre-fed flies (*t*-test, $t_{16} = 4.112$; $p = 0.0008$; $n \geq 8$). Means are ± SEM; statistical test: one-way ANOVA; n.s.: $p \geq 0.05$; *$p < 0.05$; ***$p < 0.001$ in comparison between two groups.
